# Examining the association among fear of COVID‐19, psychological distress, and delays in cancer care

**DOI:** 10.1002/cam4.4391

**Published:** 2021-11-29

**Authors:** Nicole E. Caston, Valerie M. Lawhon, Karen Lisa Smith, Kathleen Gallagher, Rebekah Angove, Eric Anderson, Alan Balch, Andres Azuero, Chao‐Hui Sylvia Huang, Gabrielle B. Rocque

**Affiliations:** ^1^ Division of Hematology and Oncology University of Alabama at Birmingham (UAB) Birmingham Alabama USA; ^2^ Johns Hopkins University School of Medicine Baltimore Maryland USA; ^3^ Patient Advocate Foundation Hampton Virginia USA; ^4^ Division of Gerontology, Geriatrics and Palliative Care University of Alabama at Birmingham (UAB) Birmingham Alabama USA

**Keywords:** care disruption, COVID‐19, COVID‐19 fear, mental health, oncology, psychological distress

## Abstract

**Background:**

Given the high risk of COVID‐19 mortality, patients with cancer may be vulnerable to fear of COVID‐19, adverse psychological outcomes, and health care delays.

**Methods:**

This longitudinal study surveyed the pandemic's impact on patients with cancer (*N*= 1529) receiving Patient Advocate Foundation services during early and later pandemic. Generalized estimating equation with repeated measures was conducted to assess the effect of COVID‐19 on psychological distress. Logistic regression with repeated measures was used to assess the effect of COVID‐19 on any delays in accessing health care (e.g., specialty care doctors, laboratory, or diagnostic testing, etc.).

**Results:**

Among 1199 respondents, 94% considered themselves high risk for COVID‐19. Respondents with more fear of COVID‐19 had a higher mean psychological distress score (10.21; 95% confidence intervals [CI] 9.38–11.03) compared to respondents with less fear (7.55; 95% CI 6.75–8.36). Additionally, 47% reported delaying care. Respondents with more fear of COVID‐19 had higher percentages of delayed care than those with less (56; 95% CI 39%–72% vs. 44%; 95% CI 28%–61%). These relationships persisted throughout the pandemic. For respondents with a COVID‐19 diagnosis in their household (*n* = 116), distress scores were similar despite higher delays in care (58% vs. 27%) than those without COVID‐19.

**Conclusions:**

Fear of COVID‐19 is linked to psychological distress and delays in care among patients with cancer. Furthermore, those who are personally impacted see exacerbated cancer care delays. Timely psychosocial support and health care coordination are critical to meet increased care needs of patients with cancer during the COVID‐19 pandemic.

## Graphical abstract

Fear of COVID‐19 is associated with psychological distress and delay in cancer care. Timely psychosocial support and health care coordination are needed to meet increased care needs of patient with cancer during the pandemic.

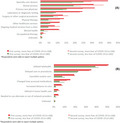

## 1 BACKGROUND

Prior to the COVID‐19 pandemic, patients with cancer were at higher risk for psychological distress compared to patients without cancer.^1^ During the COVID‐19 pandemic, patients with cancer had potential for further distress and adverse mental health impacts related to delayed or missed appointments and treatments during the pandemic.^2^, ^3^ Furthermore, the psychological sequelae of COVID‐19 mitigation strategies, such as social distancing and increased hygiene practices, can further exacerbate these patients’ emotional well‐being. Zhu and colleagues found that negative emotions and alienation due to social distancing are predictors for post‐traumatic stress disorder.^4^ A growing body of literature has found higher frequency of mental health symptoms (e.g., anxiety, depression, distress, post‐traumatic stress disorder) among patients with cancer during the COVID‐19 pandemic.^5^, ^6^, ^7^ For instance, Ng and colleagues reported that 66% of patients with cancer reported a high level of fear of COVID‐19.^8^ Furthermore, qualitative data from the early pandemic highlighted fears related to potential infection as well as risk of exposure while receiving health care.^9^

Psychological distress, when not addressed, is linked to poor survivorship outcomes including worsening symptoms and poor quality of life in patients with cancer.^10^ Anxious repetitive thoughts are associated with poor quality of life, early treatment drop‐out, and substance misuse.^11^ Prior cross‐sectional or retrospective studies have characterized mental health impacts of the coronavirus on the general population or medical staff.^12^, ^13^, ^14^ Additionally, due to the changing nature of the pandemic and pandemic‐related restrictions, and regulations, self‐reported mental health changed throughout the pandemic.^15^ However, limited data exist on cancer populations with limited financial resources, which have disproportionately experienced COVID‐19 infection and adverse outcomes from the disease when compared to the general population.^16^, ^17^, ^18^, ^19^ To bridge the gap in the literature, our study aims to evaluate the association between fear of COVID‐19, mental health outcomes, and delays in care delivery for underserved patients with cancer during the pandemic.

## 2 METHODS

### 2.1 Study design and sample

This observational, longitudinal study uses data from nationwide surveys (Figure S1) distributed by Patient Advocate Foundation (PAF), a nonprofit organization that provides case management and various forms of financial aid to patients with a diagnosis of a chronic illness within the United States.^20^ This population differs from the general population as they have at least one comorbidity and are low‐resourced. For this analysis, the subset of patients with a diagnosis of cancer (*N* = 1529) who completed the survey were included. Exclusion criteria for analysis included caregiver proxy response, missing age, sex, cancer type, or 12‐digit Federal Information Processing Standards (FIPS) code denoting census tract. Data collection were conducted at two time points: (1) 20 May to 11 July 2020 (early pandemic) and (2) 2 December to 23 December 2020 (later pandemic). Surveys were distributed via e‐mail to those who received PAF services from July 2019 to April 2020; individuals received up to three reminder e‐mails. The surveys contained questions that focused on individual experiences with COVID‐19 and the psychological, emotional, physical, and material effects from the pandemic. This study was approved by the University of Alabama at Birmingham Institutional Review Board (IRB: 300002721) prior to commencing this study.

### 2.2 Variables

The following variables were selected conceptually as they had a potential impact on the relationship between fear of COVID‐19 on both psychological distress and delayed care.

### 2.3 Patient and clinical characteristics

#### 2.3.1 Patient characteristics

Employment status and education level were self‐reported. Age, sex, race and ethnicity, region, annual household income, household size, marital status, and FIPS code were abstracted from the PAF database. Area Deprivation Index (ADI) and Rural‐Urban Commuting Area (RUCA) values were determined using respondent's census tract 12‐digit FIPS code. ADI scores range from 0 to 100; with scores in the top 15% considered most socioeconomically disadvantaged and the lower 85% of scores considered least disadvantaged, as recommended by Kind et al.^21^, ^22^ RUCA scores were used to determine rurality or urbanicity of respondent's residence.^23^

#### 2.3.2 Clinical characteristics

Cancer type was self‐reported by respondents. Cancer types were grouped into common categories (breast, gastrointestinal, genitourinary, gynecological, hematology, and other). Respondents self‐reported comorbidities from a list for which they are in active or should be in treatment; comorbidities were categorized into either 0 (cancer only), 1–2, or 3+ number of comorbidities. Respondents also self‐reported if they considered themselves to be at higher risk for severe illness if they were to be infected with COVID‐19.

#### 2.3.3 Care delivery characteristics

Survey respondents reported ability to access care and change in access. The type of care that was disrupted included specialty/primary care provider/other; surgery/labs; other services; no disruption. How respondents changed the way they accessed care included cancellation/delay; out‐of‐network/alternative provider; increased distance traveled; online/telehealth/home health; no changes. Respondents were able to report multiple options.

#### 2.3.4 COVID‐19 diagnosis

Respondents self‐reported a COVID‐19 diagnosis for either themselves, a household member, or both in either the first or second survey. The first survey after reporting a COVID‐19 diagnosis was used for analysis.

#### 2.3.5 COVID‐19‐specific characteristics

Using publicly available data, perceived risk of COVID‐19 was determined using the weekly average of COVID‐19 cases per 100,000 for each respondent's county the week prior to survey submission.^24^ Respondents were asked questions regarding their pandemic hygiene practice from the previous month, including washing hands frequently, avoiding touching the face, covering coughs, and avoiding touched surfaces in public places.^25^ Responses were categorized on a four‐point scale: all of the time (1), most of the time (2), sometimes (3), rarely (4). The degree of stress from social distancing recommendations was self‐reported using a four‐point scale: a lot (1), somewhat (2), a little (3), not at all (4).^26^

### 2.4 Study exposure and outcomes

#### 2.4.1 Fear of COVID‐19 (exposure)

The Fear of COVID‐19 Scale (FCV‐19S) was used to determine fear of COVID‐19. FCV‐19S has been validated using the Hospital Anxiety and Depression Scale and the Perceived Vulnerability to Disease Scale.^27^ FCV‐19S features seven questions with responses on a five‐point scale: strongly disagree (1), disagree (2), neutral (3), agree (4), strongly agree (5). Scores range from 7 to 35; with higher scores representing more fear. Fear of COVID‐19 was split between those with scores ≥22 (more fear of COVID‐19) and <22 (less fear of COVID‐19), as a score of 22 was consistent with “neutral” for all answers. According to a distribution‐based methodology for assessing minimally important differences, a change in score between the first and second surveys exceeding ½ standard deviation represented a change in fear of COVID‐19.^28^

#### 2.4.2 Psychological distress (outcome)

Psychological distress was determined using a four‐item questionnaire by Holingue and colleagues assessing psychological symptoms for the past 7 days.^29^ The questionnaire asks if respondent (1) felt nervous, anxious, or on edge, (2) felt depressed, (3) felt lonely, and (4) had trouble sleeping. Responses are on a four‐point scale: rarely or none of the time (<1 day); some or a little of the time (1–2 days); occasionally or a moderate amount of time (3–4 days); most or all of the time (5–7 days); scores range from 4 to 16 with higher scores denoting higher distress.

#### 2.4.3 Delayed care (outcome)

Respondents were asked, “Thinking about the health condition(s) you just mentioned, have you delayed care or had your treatment interrupted due to the COVID‐19 pandemic?” Delayed care due to COVID‐19 restrictions was categorized as patient election, hospital or provider election, income loss, insurance loss or difficulty accessing medications, or other medical care. Delayed care was dichotomized as any versus none for modeling. Concern of potential long‐term health issues due to delay in care or treatment was assessed on a five‐point scale: extremely concerned (1), very concerned (2), moderately concerned (3), slightly concerned (4), not concerned at all (5).

### 2.5 Statistical methods

Median and interquartile ranges were reported for continuous variables, and frequencies and percentages were reported for categorical variables by time point (early or later in the pandemic). For individuals (*n* = 12) with missing items in the psychological distress questionnaire, we used item mean imputation.^30^ Due to the changing nature of the pandemic, we asked respondents their fear of COVID‐19, delayed care, and distress at the second survey to understand how these factors may have changed. We estimated means and 95% confidence intervals (CI) using a generalized estimating equation with repeated measures model to assess the effect of fear of COVID‐19 on psychological distress early and later in the pandemic. We estimated percentages and 95% CI using a logistic regression with repeated measures model to assess the effect of fear of COVID‐19 on delayed care early and later in the pandemic. We also performed the same models above but using a lagged approached with our exposure fear of COVID‐19 assessed at first survey and outcomes of delayed care and distress assessed at the second survey. All models were adjusted for age, sex, race and ethnicity, region, annual household income, household size, marital status, employment status, ADI category, RUCA category, cases per 100,000, cancer type, and number of comorbidities. Analyses were performed using SAS© software, version 9.4 (SAS Institute).

## 3 RESULTS

### 3.1 Fear of COVID‐19, psychological distress, and delayed care

Among the 1529 patients with cancer completing the first survey, 1199 (78%) were eligible for inclusion; 22% were excluded due to missing data. The majority of respondents were female (72%), within the 56–75 age group (55%); 23% were Black. Most respondents had 1–2 comorbidities (34%; Table 1). The majority (94%) considered themselves at risk for COVID‐19 and reported practicing pandemic hygiene all of the time (77%). They also reported that, within the past month, social distancing behaviors affected their mental health a lot (25%), somewhat (30%), a little (25%), not at all (20%), and unknown (0.5%).

**TABLE 1:** Respondent demographic, clinical, and pandemic characteristics by survey response

	All respondents who completed first survey	Second survey respondents who completed both first and second survey	Respondent and/or household member with COVID−19 in either survey
	*n* = 1199	*n* = 448	*n* = 116
	*n* (%)	*n* (%)	*n* (%)
Age group			
19–35	47 (3.9)	15 (3.4)	5 (4.3)
36–55	415 (34.6)	153 (34.2)	49 (42.2)
56–75	655 (54.6)	256 (57.1)	55 (47.4)
>75	82 (6.8)	24 (5.4)	7 (6.0)
Sex			
Female	868 (72.4)	322 (71.9)	79 (68.1)
Male	331 (27.6)	126 (28.1)	37 (31.9)
Race and ethnicity			
Black	279 (23.2)	84 (18.8)	46 (39.7)
Hispanic/Latino	92 (7.7)	25 (5.6)	9 (7.8)
Other	60 (5.0)	25 (5.6)	7 (6.0)
White	719 (60.0)	298 (66.5)	49 (42.2)
Unknown	49 (4.1)	16 (3.6)	5 (4.3)
Region			
Midwest	222 (18.5)	85 (19.0)	24 (20.7)
Northeast	157 (13.1)	69 (15.4)	45 (38.8)
South	622 (51.9)	211 (47.1)	34 (29.3)
West	198 (16.5)	83 (18.5)	13 (11.2)
Annual household income			
≤$23,999	386 (32.2)	127 (28.4)	36 (31.0)
$24,000–$47,999	495 (41.3)	189 (42.2)	48 (41.4)
$48,000–$71,999	185 (15.4)	76 (17.0)	12 (10.3)
$72,000–$95,999	66 (5.5)	30 (6.7)	7 (6.0)
$96,000–$119,999	11 (0.9)	4 (0.9)	1 (0.9)
≥$120,000	46 (3.8)	22 (4.9)	6 (5.2)
Unknown	10 (0.8)	NA	6 (5.2)
Household size			
1	327 (27.3)	124 (27.7)	25 (21.6)
2	457 (38.1)	167 (37.3)	37 (31.9)
3	190 (15.9)	69 (15.4)	27 (23.3)
4+	221 (18.4)	87 (19.4)	27 (23.3)
Unknown	4 (0.3)	1 (0.2)	0
Marital status			
Divorced/separated/widow	305 (25.4)	108 (24.1)	36 (31.0)
Married, or living as married	530 (44.2)	214 (47.8)	44 (37.9)
Single	326 (27.2)	117 (26.1)	35 (30.2)
Unknown	38 (3.2)	9 (2.0)	1 (0.9)
Employment status			
Disabled	452 (37.7)	152 (33.9)	42 (36.2)
Employed	257 (21.4)	97 (21.7)	38 (32.8)
Retired	333 (27.8)	139 (31.0)	21 (18.1)
Unemployed/other	157 (13.1)	60 (13.4)	15 (12.9)
Education level			
Less than high school	30 (2.5)	10 (2.2)	5 (4.3)
High school	260 (21.7)	73 (16.3)	26 (22.4)
Some college	436 (36.4)	170 (38.0)	41 (35.3)
Bachelor's degree or more	468 (39.0)	191 (42.6)	44 (37.9)
Unknown	5 (0.4)	4 (0.9)	0
Area Deprivation Index			
Most disadvantaged	142 (11.8)	43 (9.6)	16 (13.8)
Least disadvantaged	1057 (88.2)	405 (90.4)	82 (70.7)
Unknown	0	0	18 (15.5)
Rural‐urban commuting area			
Rural	137 (11.4)	51 (11.4)	9 (7.8)
Urban	1062 (88.6)	397 (88.6)	89 (76.7)
Unknown	0	0	18 (15.5)
Cancer type			
Breast	413 (34.5)	147 (32.8)	37 (31.9)
Gastrointestinal	64 (5.3)	26 (5.8)	6 (5.2)
Genitourinary	73 (6.1)	31 (6.9)	5 (4.3)
Gynecological	29 (2.4)	12 (2.7)	2 (1.7)
Hematologic	359 (29.9)	146 (32.6)	38 (32.8)
Other	261 (21.8)	86 (19.2)	28 (24.1)
Number of comorbidities			
0 (cancer only)	480 (40.0)	177 (39.5)	43 (37.1)
1–2	409 (34.1)	153 (34.2)	37 (31.9)
3+	310 (25.9)	118 (26.3)	28 (24.1)
Unknown	0	0	8 (7.9)
Fear of COVID‐19 score, median (IQR)	20 (15‐24)	19 (15‐23)	NA
Fear of COVID‐19 groups			
More fearful of COVID‐19	464 (38.7)	166 (37.0)	NA
Less fearful of COVID‐19	735 (61.3)	282 (63.0)	NA
Change in fear of COVID‐19 scores between first and second surveys (by ½ standard deviation)			
No change	NA	253 (56.5)	NA
Decreased individual scores	NA	109 (24.3)	NA
Increased individual scores	NA	86 (19.2)	NA
Psychological distress score, median (IQR)	8 (6‐11)	8 (5‐11)	9 (7‐12) *n* = 89
Delayed care			
Patient election	153 (12.8)	68 (15.2)	15 (12.9)
Hospital or provider election	324 (27.0)	84 (18.8)	42 (36.2)
Income loss	17 (1.4)	16 (3.6)	2 (1.7)
Insurance loss	14 (1.2)	7 (1.6)	2 (1.7)
Difficulty in accessing medications or other medical care	59 (4.9)	16 (3.6)	6 (5.2)
I did not experience any delay in treatment or interruption in care	632 (52.7)	257 (57.4)	26 (22.4)
Unknown	0	0	23 (19.8)
Concern of delayed care			
Extremely concerned	124 (10.3)	38 (8.5)	27 (23.3)
Very concerned	118 (9.8)	54 (12.1)	5 (4.3)
Moderately concerned	159 (13.3)	42 (9.4)	21 (18.1)
Slightly concerned	118 (9.8)	35 (7.8)	10 (8.6)
Not concerned at all	48 (4.0)	22 (4.9)	4 (3.5)
No delay	633 (52.7)	257 (57.4)	26 (22.4)
Unknown	0	0	23 (19.8)
Total cases per 100,000, median (IQR)	39 (19‐80)	8288 (6205‐10,266)	4053 (43‐9114) *n* = 96
How often in the past month are you doing the recommended pandemic hygiene?			
All of the time	925 (77.2)	348 (77.7)	NA
Not all of the time	269 (22.4)	99 (22.1)	NA
Unknown	5 (0.4)	1 (0.2)	NA
Social distancing causing stress in the past month			
A lot	294 (24.5)	94 (21.0)	NA
Somewhat	357 (29.8)	161 (35.9)	NA
A little	304 (25.4)	98 (21.9)	NA
Not at all	238 (19.9)	93 (20.8)	NA
Unknown	6 (0.5)	2 (0.5)	NA

Note

Other race and ethnicity contains American Indian/Alaska Native, Asian, blended race, Caribbean Islander, Middle Eastern, Native Hawaiian/Other Pacific Islander. Other employment contains student and other. Other cancer type contains bone, endocrine, head & neck, lung, neurological, ocular, sarcoma, skin, thyroid, and other. Fear of COVID‐19 Scale scores range from 7 to 35; with higher scores representing more fear. Scores for the psychological distress scale range from 4 to 16 with higher scored denoting higher distress.

The median (IQR) overall score of fear of COVID‐19 was 20 (15–24); 735 (61%) respondents were categorized as having less fear of COVID‐19, while 464 (39%) respondents were categorized as having more fear of COVID‐19. The median (IQR) score of psychological distress was 8 (6–11). Forty‐seven percent of respondents reported delaying any type of care due to COVID‐19: patient election (13%), hospital or provider election (27%), income loss (1%), insurance loss (1%), or difficulty accessing medications or other medical care (5%; Table 1). For respondents with more fear of COVID‐19 and less fear of COVID‐19, the most common disruption in care was to a specialty care doctor (35% and 24%, respectively; Figure S2). The most common change in care for respondents was the utilization of telehealth for both those with more and less fear of COVID‐19 (47% and 40%, respectively; Figure S3).

### 3.2 Evolution during the pandemic

**FIGURE 1:**
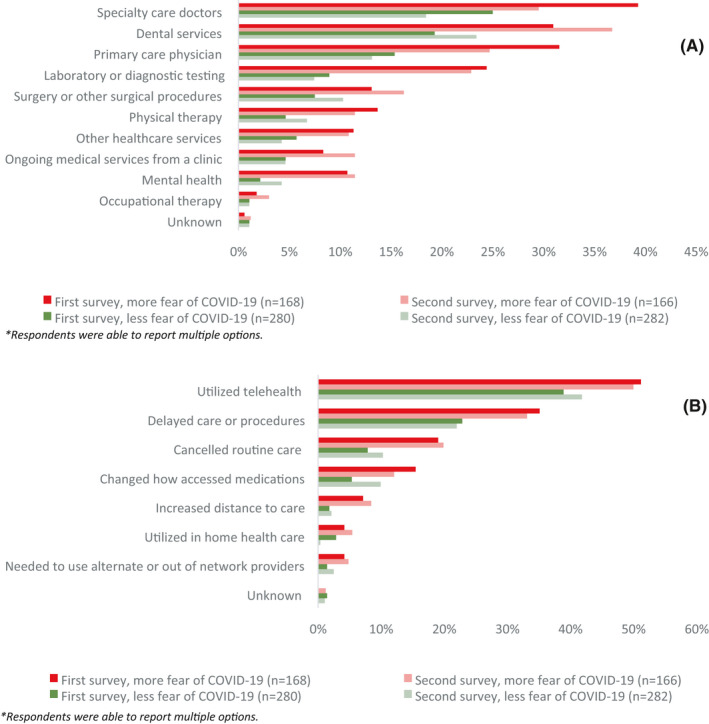
(A) Disruption in ability to access health care by level of fear during early and later COVID‐19 pandemic. (B) Change in care by level of fear early versus later COVID‐19 pandemic. Of note, respondents were able to report multiple options

Of the 1529 individuals who completed the first survey, 448 (29%) respondents completed the second survey. Demographic, clinical, and pandemic characteristics of this subset were similar to the overall cohort (Table 1). On the second survey, 253 (56%) respondents’ fear of COVID‐19 scores did not change, while 109 (24%) decreased scores, and 86 (19%) increased scores. The proportion of people practicing pandemic hygiene all of the time was stable from first to second survey (80% vs. 78%). Later in the pandemic, respondent's median (IQR) fear of COVID‐19 scores was 19 (15–23); median (IQR) psychological distress scores was 8 (5–11); and 42.6% of respondents reported delay in care (Table 1). Respondents with more fear of COVID‐19 reported more disruption in ability to access care when compared to respondents with less fear of COVID‐19 at both time points. For both respondents with more and less fear of COVID‐19, disruption in ability to access specialty care doctors decreased between the two surveys (39% to 30% and 25% to 18%, respectively). A decrease in disruption to access care was also seen for primary care physicians and laboratory or diagnostic testing. However, for respondents with both more and less fear of COVID‐19, there was an increase in disruption to accessing dental services (31%–37% and 19%–23%, respectively) and surgery or other surgical procedures (13%–16% and 8%–10%, respectively; Figure 1). Respondents with more fear of COVID‐19 reported more change in the way they accessed care when compared to respondents with less fear of COVID‐19, at both time points. Those with more fear of COVID‐19 used telehealth more than those with less fear of COVID‐19, both early (51% vs. 39%) and later in the pandemic (50% vs. 42%; Figure 1).

In adjusted models of respondents who completed both surveys, respondents with more fear of COVID‐19 early in the pandemic had a higher mean psychological distress score when compared to respondents with less fear of COVID‐19 (10.21; 95% CI 9.38–11.03 vs. 7.55; 95% CI 6.75–8.36). Similar findings were observed later in the pandemic (9.93; 95% CI 8.99–10.86 vs. 7.83; 95% CI 6.95–8.71). Early in the pandemic, respondents with more fear of COVID‐19 had higher percentages of delayed care (56%; 95% CI 39%–72%) when compared to those with less fear of COVID‐19 (44%; 95% CI 28%–61%). This relationship was consistent later in the pandemic: those with more fear of COVID‐19 had higher percentages of delayed care (55%; 95% CI 35%–73%) when compared to those with less fear of COVID‐19 (38%; 95% CI 22%–57%; Table 2). Crude model results can be found in Table S1. Full model results can be found in Tables S2 and S3.

**TABLE 2:** Model‐estimated means and 95% confidence intervals of psychological distress and model‐estimated percentages and 95% confidence intervals of delayed care early pandemic (*n* = 1199), later pandemic (*n* = 448), and lagged approach (*n* = 448)

	Early pandemic model‐estimated means (95% CI)	Later pandemic model‐estimated means (95% CI)	Lagged approach model‐estimated means (95% CI)
	(*n* = 1199)	(*n* = 448)	(*n* = 448)
Distress scale			
Fear of COVID‐19 Scale			
More fear of COVID‐19	10.21 (9.38–11.03)	9.93 (8.99–10.86)	10.79 (9.04–12.54)
Less fear of COVID‐19	7.55 (6.75–8.36)	7.83 (6.95–8.71)	8.26 (7.04–9.47)

	Early pandemic model‐estimated percentages (95% CI)	Later pandemic model‐estimated percentages (95% CI)	Lagged approach model‐estimated percentages (95% CI)
	(*n* = 1199)	(*n* = 448)	(*n* = 448)
Delayed care			
Fear of COVID‐19 Scale			
More fear of COVID‐19	56.3% (39.4%–71.9%)	54.8% (35.2%–73.0%)	48.0% (20.3%–75.7%)
Less fear of COVID‐19	43.6% (28.1%–60.5%)	37.9% (22.2%–56.7%)	39.7% (11.6%–67.8%)

Note

Each model is adjusted for age, sex, race and ethnicity, region, annual household income, household size, marital status, employment status, Area Deprivation Index category, Rural‐Urban Commuting Code category, cases per 100,000, cancer type, and number of comorbidities. Full model results can be found in Tables S2–S5. Scores for the psychological distress scale range from 4 to 16 with higher scores denoting higher distress.

Abbreviation: CI, confidence intervals.

Using the lagged approach, similar findings were seen within the early and later pandemic: those with more fear of COVID‐19 had higher mean psychological distress scores and higher percentages of delayed care than those with less fear of COVID‐19 (Table 2). Full model results for the lagged approached can be found in Tables S4 and S5.

### 3.3 Experience of patients with COVID‐19

**FIGURE 2:**
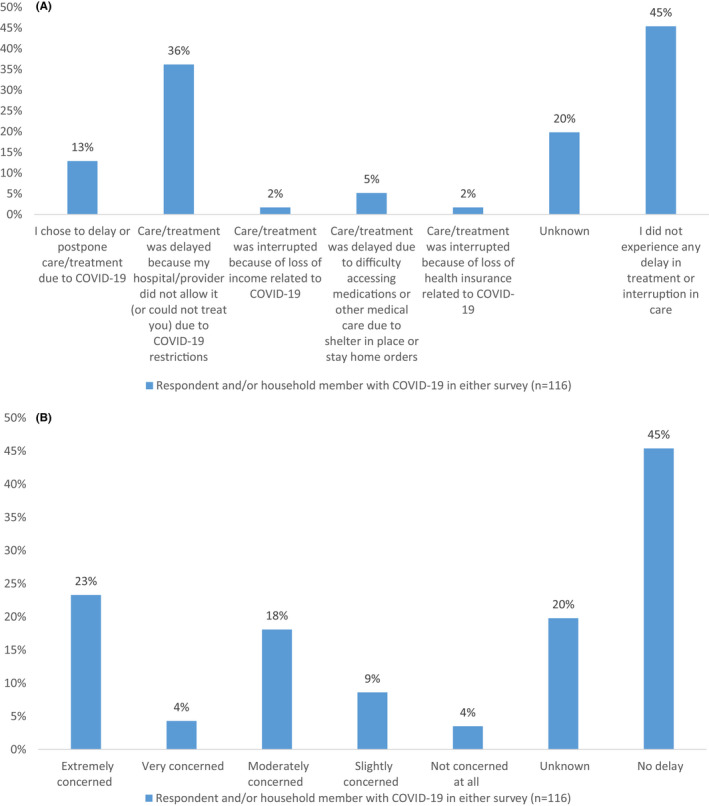
(A) Comparison of reason for delay in care due to COVID‐19 pandemic for respondents and/or household member diagnosed with COVID‐19 (*n* = 116). (B) To what extent respondents are worried about long‐term health issues related to delay in care for respondents and/or household member diagnosed with COVID‐19 (*n* = 116)

Hundred and sixteen respondents who completed the survey reported themselves and/or a household member having a diagnosis of COVID‐19. These patients were predominantly female (68%), White (42%), and aged 56–75 (47%; Table 1). Respondents who reported a COVID‐19 diagnosis experienced a median (IQR) psychological distress scores of 9 (7–12), similar to those without COVID‐19 who had a median score of 8. They experienced higher rates of delays in care (58% vs. 27%) than those without COVID‐19 in the first survey, with greater delays due to hospital or provider election (36% vs. 27%, respectively; Table 1). Additionally, 23% of the respondents were extremely concerned about long‐term health effects related to the delay (Figure 2).

## 4 DISCUSSION

This study demonstrates that fear of COVID‐19 is common, with 39% of patients reporting high levels of fear in a population that is financially constrained with substantial risk of infection, morbidity, and mortality. Over half reported detrimental effect of social distancing on their mental health, a finding consistent with previous research shows that long periods of social isolation have adverse effects on a person's mental health.^31^ The psychological outcomes from the pandemic are not without impact, given the strong association observed between fear of COVID‐19 and both mental health and delays in care.

To our knowledge, this is one of the first studies assessing the relationship between changes in healthcare delivery on mental health outcomes during the COVID‐19 pandemic. Approximately 50% of respondents reported delays in healthcare delivery, and the vast majority with delays reported concern about impact of delays on long‐term health. Reasons for delays were multi‐faceted, but <20% were attributed to patient election, indicating provider‐, and healthcare‐system level interruptions in care. Our data are consistent with previously published findings indicating decreased healthcare utilization among patients with cancer during the pandemic. In a study of US Medicare beneficiaries, Patt et al. reported decreases in the number of physician encounters, screening procedures, biopsies, and cancer surgeries.^3^

The proportion of patients who had a delay or missed health care service was higher during the early pandemic. This is consistent with adherence to guidelines published early in the pandemic suggesting that routine cancer screening and nonurgent cancer surgeries be delayed.^32^, ^33^ However, as the pandemic persisted and knowledge about safe delivery of cancer care expanded, restrictions were lifted and oncology providers resumed a more standard practice over time. Although concerns about the potential risks associated with immunosuppression from cancer therapy were raised early in the pandemic, data now indicate that, with the exception of hematologic malignancies, chemotherapy can be safely administered to patients with cancer during the pandemic.^34^, ^35^, ^36^ Despite this knowledge, our data highlight that challenges remain that impact patient care  later in the pandemic and that these deviations are concentrated among patients who have higher fear of COVID‐19. These delays were markedly worse for patients reporting an infection with the COVID‐19 virus within their household. While providers may be modifying treatment schedules or plans based on patients' request, our data suggest that these modifications are associated with adverse psychological outcomes. Thus, patients who have a patient‐, provider‐, or system‐driven delay should be screened for psycho‐oncological distress. NCCN Distress Management Guidelines recommends that “distress should be recognized, monitored, documented, and treated promptly at all stages of disease and in all settings.”^37^ Findings from this study highlights the need for offering timely screening and referrals to meet patients’ psychosocial care needs during the COVID‐19 pandemic.

This study has several limitations. Due to the novel nature of the pandemic, the fear of COVID‐19 scale lacks literature evaluating psychometrics. However, the face validity as well as the strong correlation with the mental health scale enhances the reliability. It is important to note that patients with higher fear of COVID‐19 may have specific risk factors that predispose them to adverse outcomes. However, given that all patients had a cancer diagnosis and socioeconomic characteristics of this population, the vast majority of the patients would be categorized as high risk. Since our population contains individuals with cancer who are under resourced and seeking health services, it is not representative of a general population. In addition, this population of under‐resourced individuals differs from a general cancer population as cancer affects individuals of all backgrounds. However, our population is similar to samples of under‐resourced patients with cancer receiving financial aid.^38^ A study using SEER data by Clegg et al. found that cancer incidence and socioeconomic statuses (SES) differed among types of cancer: higher SES was found to be associated with prostate and breast cancer incidence, while lower SES was found to be associated with lung and colorectal cancer incidence.^39^

The lack of detailed information about current cancer treatment limits ability to evaluate impact by treatment type or phase of care nor if cancer care was delayed specifically. Patients with underlying distress, or who experienced care delays, may be more susceptible to fears related to COVID‐19 and vice versa. Also, respondents may have been unaware of county‐level cases of COVID‐19 at the time of survey competition. Demographic information on nonrespondent individuals with cancer is unavailable due to the inability to capture cancer diagnosis for those who did not initiate the survey. The response rate on the second survey was low and demographics between those who completed first survey, both surveys, and first were similar and can be found in Table S6. Lastly, all information was self‐reported by respondents, which may result in information bias.

## 5 CONCLUSIONS

Fear of COVID‐19 is linked to psychological distress and delays or modifications in care among patients with cancer. Furthermore, for patients with cancer who are personally impacted by COVID‐19, care delays are exacerbated. Timely psycho‐oncological support and care coordination will be critical to meet the increased mental health and access to care needs of patients with cancer during the COVID‐19 pandemic. Future studies are needed to evaluate the long‐term impact of COVID‐related fear on psychological distress and care delivery in patients with cancer, and identify system‐wide strategies to provide timely distress management and improve care delivery.

## CONFLICT OF INTEREST

Dr. Rocque received research funding from Genentech, Pfizer, and Carevive and consulting fees from Genentech and Pfizer. Dr. Smith received research funding from Pfizer, and her spouse has stock in ABT Laboratories and Abbvie.

## ETHICS STATEMENT

This study was approved by the University of Alabama at Birmingham Institutional Review Board (IRB: 300002721) prior to commencing this study.

## Supporting information

Supplementary Material

## Data Availability

The data that support the findings of this study are available from the corresponding author upon reasonable request.
