# Supplementary material for: Examining the association among fear of COVID‐19, psychological distress, and delays in cancer care
Source: Cancer Med. 2021 Nov 29;10(24):8854–65. doi: 10.1002/cam4.4391 (PMC8683527; doi:10.1002/cam4.4391)
Supplement: Supplementary file 1 — Supplementary Material [file CAM4-10--s001.docx]

**Supplemental Index**

1. Tables
2. Figures

Supplemental Table 1. Crude beta coefficients, means, probabilities, and corresponding 95% confidence intervals of psychological distress and delayed care for both early and later pandemic

|  | Beta Coefficients  (95% CI) | Means  (95% CI) |
| --- | --- | --- |
|  | **(n=1199)** |  |
| **Distress scale** |  |  |
| Intercept | 7.49 (7.19–7.79) |  |
| *Fear of COVID-19 Scale* |  |  |
| More fear of COVID-19 | 2.33 (1.83–2.83) |  |
| Less fear of COVID-19 | Ref. |  |
| *Time* |  |  |
| Early pandemic | -0.08 (-0.37-0.20) |  |
| Later pandemic | Ref. |  |
| *Fear of COVID-19 Scale*Time* |  |  |
| More fear of COVID-19*early | 0.63 (0.10-1.16) | 10.37 (10.09-10.65) |
| More fear of COVID-19*later | Ref. | 9.82 (9.40-10.24) |
| Less fear of COVID-19*early | Ref. | 7.41 (7.20-7.61) |
| Less fear of COVID-19*later | Ref. | 7.49 (7.19-7.79) |
|  | Beta Coefficients  (95% CI) | Probabilities  (95% CI) |
|  | **(n=1199)** |  |
| **Delayed care** |  |  |
| Intercept | -0.62 (-0.85–-0.39) |  |
| *Fear of COVID-19 Scale* |  |  |
| More fear of COVID-19 | 0.79 (0.42–1.16) |  |
| Less fear of COVID-19 | Ref. |  |
| *Time* |  |  |
| Early pandemic | 0.28 (0.03-0.53) |  |
| Later pandemic | Ref. |  |
| *Fear of COVID-19 Scale*Time* |  |  |
| More fear of COVID-19*early | -0.19 (-0.60-0.22) | 56% (52%-61%) |
| More fear of COVID-19*later | Ref. | 54% (47%-61%) |
| Less fear of COVID-19*early | Ref. | 42% (38%-45%) |
| Less fear of COVID-19*later | Ref. | 35% (30%-40%) |

Scores for the psychological distress scale range from 4-16 with higher scores denoting higher distress. CI=confidence intervals.

Supplemental Table 2. Model-estimated beta coefficients and 95% confidence intervals of psychological distress (n=1199).

|  | Beta Coefficients  (95% CI) |
| --- | --- |
|  | **(n=1199)** |
| **Distress scale** |  |
| Intercept | 9.79 (6.92–12.66) |
| *Fear of COVID-19 Scale* |  |
| More fear of COVID-19 | 2.07 (1.58–2.56) |
| Less fear of COVID-19 | Ref. |
| *Time* |  |
| Early in the pandemic | -0.32 (-0.89–0.25) |
| Later in the pandemic | Ref. |
| *Fear of COVID-19 Scale*Time* |  |
| More fear of COVID-19*early | 0.58 (0.06–1.10) |
| More fear of COVID-19*later | Ref. |
| Less fear of COVID-19*early | Ref. |
| Less fear of COVID-19*later | Ref. |
| *Age group* |  |
| 19-35 | 1.88 (0.75–3.02) |
| 36-55 | 1.46 (0.73–2.19) |
| 56-75 | 0.61 (0.03–1.19) |
| >75 | Ref. |
| *Sex* |  |
| Female | 0.37 (-0.06–0.80) |
| Male | Ref. |
| *Race/ethnicity* |  |
| Black | -0.65 (-1.08–-0.23) |
| Hispanic/Latino | -0.87 (-1.56–-0.19) |
| Other | -0.40 (-1.19–0.38) |
| Unknown | -0.25 (-1.02–0.52) |
| White | Ref. |
| *Region* |  |
| Midwest | -0.13 (-0.66–0.40) |
| Northeast | 0.18 (-0.40–0.77) |
| South | -0.10 (-0.56–0.35) |
| West | Ref. |
| *Annual household income* |  |
| ≤ $23,999 | 0.18 (-1.74–2.11) |
| $24,000-$47,999 | 0.25 (-1.66–2.16) |
| $48,000-$71,999 | -0.08 (-2.02–1.86) |
| $72,000-$95,999 | 0.21 (-1.81–2.24) |
| $96,000-$119,999 | -1.91 (-4.28–0.47) |
| ≥ $120,000 | 0.58 (-1.47–2.64) |
| Unknown | Ref. |
| *Household size* |  |
| 1 | -2.10 (-4.90–0.71) |
| 2 | -2.66 (-5.47–0.16) |
| 3 | -2.12 (-4.95–0.70) |
| 4+ | -2.06 (-4.92–0.79) |
| Unknown | Ref. |
| *Marital status* |  |
| Divorced/separated/widow | -0.08 (-1.04–0.88) |
| Married, or living as married | -0.73 (-1.67–0.21) |
| Single | -0.87 (-1.81–0.07) |
| Unknown | Ref. |
| *Employment status* |  |
| Disabled | 0.36 (-0.13–0.85) |
| Employed | -0.29 (-0.78–0.20) |
| Retired | -0.84 (-1.36–-0.31) |
| Unemployed/other | Ref. |
| *Education level* |  |
| Less than high school | 1.11 (-0.71–2.93) |
| High school | 0.91 (-0.51–2.33) |
| Some college | 0.64 (-0.75–2.04) |
| Bachelor's degree or more | 0.42 (-0.97–1.80) |
| Unknown | Ref. |
| *Area Deprivation Index* |  |
| Least disadvantaged | 0.16 (-0.35–0.66) |
| Most disadvantaged | Ref. |
| *Rural Urban Commuting Area* |  |
| Rural | -0.11 (-0.62–0.40) |
| Urban | Ref. |
| *Total cases per 100,000* | 0 (-0.00–0) |
| *Cancer type* |  |
| Breast | 0.12 (-0.34–0.57) |
| Gastrointestinal | 0.04 (-0.85–0.93) |
| Genitourinary | -0.25 (-0.96–0.45) |
| Gynecological | -0.30 (-1.13–0.52) |
| Hematologic | 0.04 (-0.39–0.47) |
| Other | Ref. |
| *Number of comorbidities* |  |
| 0 (cancer only) | -1.11 (-1.53–-0.68) |
| 1--2 | -0.83 (-1.25–-0.42) |
| 3+ | Ref. |

Other race/ethnicity contains American Indian/Alaska Native, Asian, blended race, Caribbean Islander, Middle Eastern, Native Hawaiian/Other Pacific Islander. Other employment contains student and other. Other cancer type contains bone, endocrine, head & neck, lung, neurological, ocular, sarcoma, skin, thyroid, and other. Fear of COVID-19 Scale scores range from 7–35; with higher scores representing more fear. Scores for the psychological distress scale range from 4–16 with higher scored denoting higher distress. CI=confidence intervals.

Supplemental Table 3. Model-estimated beta coefficients and 95% confidence intervals of delayed care (n=1199).

|  | Beta Coefficients  (95% CI) |
| --- | --- |
|  | **(n=1199)** |
| **Delayed care** |  |
| Intercept | 0.86 (-1.84–3.56) |
| *Fear of COVID-19 Scale* |  |
| More fear of COVID-19 | 0.68 (0.28–1.09) |
| Less fear of COVID-19 | Ref. |
| *Time* |  |
| Early in the pandemic | 0.24 (-0.30–0.77) |
| Later in the pandemic | Ref. |
| *Fear of COVID-19 Scale*Time* |  |
| More fear of COVID-19*early | -0.17 (-0.62–0.27) |
| More fear of COVID-19*later | Ref. |
| Less fear of COVID-19*early | Ref. |
| Less fear of COVID-19*later | Ref. |
| *Age group* |  |
| 19-35 | 0.58 (-0.20–1.36) |
| 36-55 | 0.43 (-0.13–1.00) |
| 56-75 | 0.36 (-0.10–0.81) |
| >75 | Ref. |
| *Sex* |  |
| Female | -0.46 (-0.77–-0.15) |
| Male | Ref. |
| *Race/ethnicity* |  |
| Black | -0.41 (-0.73–-0.09) |
| Hispanic/Latino | -0.29 (-0.75–0.18) |
| Other | 0.31 (-0.25–0.88) |
| Unknown | -0.10 (-0.67–0.47) |
| White | Ref. |
| *Region* |  |
| Midwest | 0.22 (-0.17–0.61) |
| Northeast | 0.49 (0.06–0.92) |
| South | 0.26 (-0.08–0.60) |
| West | Ref. |
| *Annual household income* |  |
| ≤ $23,999 | -0.57 (-2.02–0.87) |
| $24,000-$47,999 | -0.59 (-2.04–0.85) |
| $48,000-$71,999 | -0.79 (-2.25–0.67) |
| $72,000-$95,999 | -0.17 (-1.67–1.33) |
| $96,000-$119,999 | -0.89 (-2.678–0.89) |
| ≥ $120,000 | -0.77 (-2.31–0.76) |
| Unknown | Ref. |
| *Household size* |  |
| 1 | -0.58 (-2.60–1.44) |
| 2 | -0.37 (-2.40–1.65) |
| 3 | -0.18 (-2.22–1.86) |
| 4+ | -0.40 (-2.45–1.65) |
| Unknown | Ref. |
| *Marital status* |  |
| Divorced/separated/widow | 0.22 (-0.39–0.83) |
| Married, or living as married | -0.17 (-0.78–0.44) |
| Single | 0.33 (-0.28–0.93) |
| Unknown | Ref. |
| *Employment status* |  |
| Disabled | 0.05 (-0.28–0.39) |
| Employed | -0.22 (-0.59–0.14) |
| Retired | -0.21 (-0.61–0.18) |
| Unemployed/other | Ref. |
| *Education level* |  |
| Less than high school | -0.19 (-2.06–1.68) |
| High school | 0.28 (-1.44–1.99) |
| Some college | 0.40 (-1.30–2.11) |
| Bachelor's degree or more | 0.64 (-1.06–2.34) |
| Unknown | Ref. |
| *Area Deprivation Index* |  |
| Least disadvantaged | -0.37 (-0.73–-0.01) |
| Most disadvantaged | Ref. |
| *Rural Urban Commuting Area* |  |
| Rural | -0.18 (-0.53–0.18) |
| Urban | Ref. |
| *Total cases per 100,000* | 0 (-0.00–0) |
| *Cancer type* |  |
| Breast | 0.08 (-0.25–0.41) |
| Gastrointestinal | -0.03 (-0.59–0.54) |
| Genitourinary | -0.51 (-1.07–0.05) |
| Gynecological | -0.36 (-1.10–0.38) |
| Hematologic | -0.12 (-0.44–0.20) |
| Other | Ref. |
| *Number of comorbidities* |  |
| 0 (cancer only) | -1.00 (-1.30–-0.70) |
| 1--2 | -0.65 (-0.95–-0.35) |
| 3+ | Ref. |

Other race/ethnicity contains American Indian/Alaska Native, Asian, blended race, Caribbean Islander, Middle Eastern, Native Hawaiian/Other Pacific Islander. Other employment contains student and other. Other cancer type contains bone, endocrine, head & neck, lung, neurological, ocular, sarcoma, skin, thyroid, and other. Fear of COVID-19 Scale scores range from 7–35; with higher scores representing more fear. CI=confidence intervals.

Supplemental Table 4. Model-estimated beta coefficients and 95% confidence intervals of psychological distress for the lagged approach (n=448).

|  | Beta Coefficients  (95% CI) |
| --- | --- |
|  | **(n=448)** |
| **Distress scale** |  |
| Intercept | 9.50 (2.41–16.59) |
| *Fear of COVID-19 Scale* |  |
| More fear of COVID-19 | 2.34 (1.74–2.94) |
| Less fear of COVID-19 | Ref. |
| *Age group* |  |
| 19-35 | 1.50 (-0.61–3.61) |
| 36-55 | 1.47 (-0.06–3.01) |
| 56-75 | 0.67 (-0.61–1.95) |
| >75 | Ref. |
| *Sex* |  |
| Female | -0.21 (-0.99–057) |
| Male | Ref. |
| *Race/ethnicity* |  |
| Black | -0.90 (-1.69–-0.10) |
| Hispanic/Latino | -0.64 (-1.92–0.63) |
| Other | 0.61 (-0.66–1.88) |
| Unknown | 1.08 (-0.48–2.64) |
| White | Ref. |
| *Region* |  |
| Midwest | 0.12 (-0.88–1.12) |
| Northeast | 0.52 (-0.47–1.51) |
| South | 0.10 (-0.70–0.91) |
| West | Ref. |
| *Annual household income* |  |
| ≤ $23,999 | Ref. |
| $24,000-$47,999 | 0.52 (-0.19–1.22) |
| $48,000-$71,999 | 0.40 (-0.53–1.34) |
| $72,000-$95,999 | 0.21 (-1.11–1.53) |
| $96,000-$119,999 | -0.15 (-3.15–2.85) |
| ≥ $120,000 | 0.35 (-1.14–1.83) |
| *Household size* |  |
| 1 | -5.84 (-11.61–-0.07) |
| 2 | -6.57 (-12.38–-0.77) |
| 3 | -5.96 (-11.80–-0.13) |
| 4+ | -6.06 (-11.94–-0.18) |
| Unknown | Ref. |
| *Marital status* |  |
| Divorced/separated/widow | 1.80 (-0.34–3.93) |
| Married, or living as married | 0.72 (-1.40–2.84) |
| Single | 0.75 (-1.41–2.91) |
| Unknown | Ref. |
| *Employment status* |  |
| Disabled | 0.25 (-0.66–1.16) |
| Employed | -0.29 (-1.25–0.68) |
| Retired | -0.93 (-1.92–0.05) |
| Unemployed/other | Ref. |
| *Education level* |  |
| Less than high school | 3.97 (0.48–7.45) |
| High school | 2.66 (-0.33–5.64) |
| Some college | 2.50 (-0.43–5.44) |
| Bachelor's degree or more | 1.99 (-0.94–4.91) |
| Unknown | Ref. |
| *Area Deprivation Index* |  |
| Least disadvantaged | 0.44 (-0.56–1.44) |
| Most disadvantaged | Ref. |
| *Rural Urban Commuting Area* |  |
| Rural | -0.20 (-1.06–0.66) |
| Urban | Ref. |
| *Total cases per 100,000* | 0.00 (-0.00–0.00) |
| *Cancer type* |  |
| Breast | 0.65 (-0.18–1.48) |
| Gastrointestinal | 0.99 (-0.36–2.34) |
| Genitourinary | -0.27 (-1.65–1.11) |
| Gynecological | 0.37 (-1.41–2.16) |
| Hematologic | -0.11 (-0.92–0.71) |
| Other | Ref. |
| *Number of comorbidities* |  |
| 0 (cancer only) | -0.43 (-1.19–0.33) |
| 1--2 | -0.07 (-0.80–0.67) |
| 3+ | Ref. |

Other race/ethnicity contains American Indian/Alaska Native, Asian, blended race, Caribbean Islander, Middle Eastern, Native Hawaiian/Other Pacific Islander. Other employment contains student and other. Other cancer type contains bone, endocrine, head & neck, lung, neurological, ocular, sarcoma, skin, thyroid, and other. Fear of COVID-19 Scale scores range from 7–35; with higher scores representing more fear. Scores for the psychological distress scale range from 4–16 with higher scored denoting higher distress. CI=confidence intervals.

Supplemental Table 5. Model-estimated beta coefficients and 95% confidence intervals of delayed care for the lagged approach (n=448).

|  | Beta Coefficients  (95% CI) |
| --- | --- |
|  | **(n=448)** |
| **Delayed care** |  |
| Intercept | 0.46 (-0.67–1.58) |
| *Fear of COVID-19 Scale* |  |
| More fear of COVID-19 | 0.08 (-0.01–0.18) |
| Less fear of COVID-19 | Ref. |
| *Age group* |  |
| 19-35 | 0.11 (-0.23–0.44) |
| 36-55 | 0.13 (-0.11–0.38) |
| 56-75 | 0.17 (-0.04-0.37) |
| >75 | Ref. |
| *Sex* |  |
| Female | -0.02 (-0.15–0.10) |
| Male | Ref. |
| *Race/ethnicity* |  |
| Black | -0.18 (-0.30–-0.05) |
| Hispanic/Latino | -0.08 (-0.28–0.12) |
| Other | 0.01 (-0.19–0.21) |
| Unknown | 0.12 (-0.12–0.37) |
| White | Ref. |
| *Region* |  |
| Midwest | 0.11 (-0.05–0.27) |
| Northeast | 0.08 (-0.08–0.23) |
| South | 0.16 (0.03–0.29) |
| West | Ref. |
| *Annual household income* |  |
| ≤ $23,999 | Ref. |
| $24,000-$47,999 | 0.05 (-0.06–0.16) |
| $48,000-$71,999 | 0.08 (-0.07–0.23) |
| $72,000-$95,999 | 0.19 (-0.02–0.39) |
| $96,000-$119,999 | -0.21 (-0.68–0.27) |
| ≥ $120,000 | -0.17 (-0.40–0.07) |
| *Household size* |  |
| 1 | -0.33 (-1.24–0.58) |
| 2 | -0.31 (-1.23–0.61) |
| 3 | -0.20 (-1.27–0.72) |
| 4+ | -0.32 (-1.25–0.61) |
| Unknown | Ref. |
| *Marital status* |  |
| Divorced/separated/widow | 0.11 (-0.23–0.45) |
| Married, or living as married | 0.06 (-0.28–0.39) |
| Single | 0.15 (-0.19–0.49) |
| Unknown | Ref. |
| *Employment status* |  |
| Disabled | 0.01 (-0.13–0.16) |
| Employed | -0.11 (-0.27–0.04) |
| Retired | -0.14 (-0.30–0.02) |
| Unemployed/other | Ref. |
| *Education level* |  |
| Less than high school | 0.21 (-0.34–0.76) |
| High school | 0.23 (-0.24–0.70) |
| Some college | 0.15 (-0.32–0.61) |
| Bachelor's degree or more | 0.16 (-0.30–0.63) |
| Unknown | Ref. |
| *Area Deprivation Index* |  |
| Least disadvantaged | -0.11 (-0.27–0.05) |
| Most disadvantaged | Ref. |
| *Rural Urban Commuting Area* |  |
| Rural | -0.04 (-0.18–0.10) |
| Urban | Ref. |
| *Total cases per 100,000* | 0.00 (-0.00–0.00) |
| *Cancer type* |  |
| Breast | 0.10 (-0.03–0.24) |
| Gastrointestinal | 0.35 (0.14–0.57) |
| Genitourinary | 0.06 (-0.15–0.28) |
| Gynecological | 0.04 (-0.25–0.32) |
| Hematologic | 0.09 (-0.04–0.22) |
| Other | Ref. |
| *Number of comorbidities* |  |
| 0 (cancer only) | -0.17 (-0.29–-0.05) |
| 1--2 | -0.19 (-0.31–-0.07) |
| 3+ | Ref. |

Other race/ethnicity contains American Indian/Alaska Native, Asian, blended race, Caribbean Islander, Middle Eastern, Native Hawaiian/Other Pacific Islander. Other employment contains student and other. Other cancer type contains bone, endocrine, head & neck, lung, neurological, ocular, sarcoma, skin, thyroid, and other. Fear of COVID-19 Scale scores range from 7–35; with higher scores representing more fear. CI=confidence intervals.

Supplemental Table 6. Respondent demographic, clinical, and pandemic characteristics by all respondents who completed first survey (n=1199), respondents who completed both surveys (n=448), and respondents who completed the first survey only (n=751)

|  | All respondents who completed first survey | Second survey respondents who completed both first and second survey | Respondents who completed first survey only |
| --- | --- | --- | --- |
|  | n=1199 | n=448 | n=751 |
|  | n (%) | n (%) | n (%) |
| **Age group** |  |  |  |
| 19-35 | 47 (3.9) | 15 (3.4) | 32 (4.3) |
| 36-55 | 415 (34.6) | 153 (34.2) | 262 (34.9) |
| 56-75 | 655 (54.6) | 256 (57.1) | 399 (53.1) |
| >75 | 82 (6.8) | 24 (5.4) | 58 (7.7) |
| **Sex** |  |  |  |
| Female | 868 (72.4) | 322 (71.9) | 546 (72.7) |
| Male | 331 (27.6) | 126 (28.1) | 205 (27.3) |
| **Race/ethnicity** |  |  |  |
| Black | 279 (23.2) | 84 (18.8) | 195 (26.0) |
| Hispanic/Latino | 92 (7.7) | 25 (5.6) | 67 (8.9) |
| Other | 60 (5.0) | 25 (5.6) | 35 (4.7) |
| White | 719 (60.0) | 298 (66.5) | 421 (56.1) |
| Unknown | 49 (4.1) | 16 (3.6) | 33 (4.4) |
| **Region** |  |  |  |
| Midwest | 222 (18.5) | 85 (19.0) | 137 (18.2) |
| Northeast | 157 (13.1) | 69 (15.4) | 88 (11.7) |
| South | 622 (51.9) | 211 (47.1) | 411 54.7) |
| West | 198 (16.5) | 83 (18.5) | 115 (15.3) |
| **Annual household income** |  |  |  |
| ≤ $23,999 | 386 (32.2) | 127 (28.4) | 259 (34.5) |
| $24,000-$47,999 | 495 (41.3) | 189 (42.2) | 306 (40.8) |
| $48,000-$71,999 | 185 (15.4) | 76 (17.0) | 109 (14.5) |
| $72,000-$95,999 | 66 (5.5) | 30 (6.7) | 36 (4.8) |
| $96,000-$119,999 | 11 (0.9) | 4 (0.9) | 7 (0.9) |
| ≥ $120,000 | 46 (3.8) | 22 (4.9) | 24 (34.5) |
| Unknown | 10 (0.8) | NA | 10 (1.3) |
| **Household size** |  |  |  |
| 1 | 327 (27.3) | 124 (27.7) | 203 (27.0) |
| 2 | 457 (38.1) | 167 (37.3) | 290 (38.6) |
| 3 | 190 (15.9) | 69 (15.4) | 121 (16.1) |
| 4+ | 221 (18.4) | 87 (19.4) | 134 (17.8) |
| Unknown | 4 (0.3) | 1 (0.2) | 3 (0.4) |
| **Marital status** |  |  |  |
| Divorced/separated/widow | 305 (25.4) | 108 (24.1) | 197 (26.2) |
| Married, or living as married | 530 (44.2) | 214 (47.8) | 316 (42.1) |
| Single | 326 (27.2) | 117 (26.1) | 209 (27.8) |
| Unknown | 38 (3.2) | 9 (2.0) | 29 (3.9) |
| **Employment status** |  |  |  |
| Disabled | 452 (37.7) | 152 (33.9) | 279 (37.2) |
| Employed | 257 (21.4) | 97 (21.7) | 159 (21.2) |
| Retired | 333 (27.8) | 139 (31.0) | 200 (26.6) |
| Unemployed/other | 157 (13.1) | 60 (13.4) | 113 (15.1) |
| **Education level** |  |  |  |
| Less than high school | 30 (2.5) | 10 (2.2) | 20 (2.7) |
| High school | 260 (21.7) | 73 (16.3) | 187 (24.9) |
| Some college | 436 (36.4) | 170 (38.0) | 266 (35.4) |
| Bachelor’s degree or more | 468 (39.0) | 191 (42.6) | 277 (36.9) |
| Unknown | 5 (0.4) | 4 (0.9) | 1 (0.1) |
| **Area Deprivation Index** |  |  |  |
| Most disadvantaged | 142 (11.8) | 43 (9.6) | 99 (13.2) |
| Least disadvantaged | 1057 (88.2) | 405 (90.4) | 625 (86.8) |
| **Rural-Urban Commuting Area** |  |  |  |
| Rural | 137 (11.4) | 51 (11.4) | 86 (11.5) |
| Urban | 1062 (88.6) | 397 (88.6) | 665 (88.5) |
| **Cancer type** |  |  |  |
| Breast | 413 (34.5) | 147 (32.8) | 266 (35.4) |
| Gastrointestinal | 64 (5.3) | 26 (5.8) | 38 (5.1) |
| Genitourinary | 73 (6.1) | 31 (6.9) | 42 (5.6) |
| Gynecological | 29 (2.4) | 12 (2.7) | 17 (2.3) |
| Hematologic | 359 (29.9) | 146 (32.6) | 213 (28.4) |
| Other | 261 (21.8) | 86 (19.2) | 175 (23.3) |
| **Number of comorbidities** |  |  |  |
| 0 (cancer only) | 480 (40.0) | 177 (39.5) | 303 (40.4) |
| 1-2 | 409 (34.1) | 153 (34.2) | 256 (34.1) |
| 3+ | 310 (25.9) | 118 (26.3) | 192 (25.6) |
| **Fear of COVID-19 score, median (IQR)** | 20 (24-15) | 19 (23-15) |  |
| **Fear of COVID-19 groups** |  |  |  |
| More fearful of COVID-19 | 464 (38.7) | 166 (37.0) | 296 (39.4) |
| Less fearful of COVID-19 | 735 (61.3) | 282 (63.0) | 455 (60.6) |
| **Change in fear of COVID-19 scores between first and second surveys (by ½ standard deviation)** |  |  |  |
| No change | NA | 253 (56.5) | NA |
| Decreased individual scores | NA | 109 (24.3) | NA |
| Increased individual scores | NA | 86 (19.2) | NA |
| **Psychological distress score, median (IQR)** | 8 (11-6) | 8 (11-5) |  |
| **Delayed care** |  |  |  |
| Patient election | 153 (12.8) | 68 (15.2) | 92 (12.3) |
| Hospital or provider election | 324 (27.0) | 84 (18.8) | 198 (26.4) |
| Income loss | 17 (1.4) | 16 (3.6) | 12 (1.6) |
| Insurance loss | 14 (1.2) | 7 (1.6) | 38 (5.1) |
| Difficulty accessing medications or other medical care | 59 (4.9) | 16 (3.6) | 10 (1.3) |
| I did not experience any delay in treatment or interruption in care | 632 (52.7) | 257 (57.4) | 401 (53.4) |
| **Concern of delayed care** |  |  |  |
| Extremely concerned | 124 (10.3) | 38 (8.5) | 79 (10.5) |
| Very concerned | 118 (9.8) | 54 (12.1) | 67 (8.9) |
| Moderately concerned | 159 (13.3) | 42 (9.4) | 104 (13.9) |
| Slightly concerned | 118 (9.8) | 35 (7.8) | 74 (9.9) |
| Not concerned at all | 48 (4.0) | 22 (4.9) | 26 (3.5) |
| No delay | 633 (52.7) | 257 (57.4) | 401 (53.4) |
| **Total cases per 100,000, median (IQR)** | 39 (80-19) | 8,288 (10,266-6,205) |  |
| **How often in the past month are you doing the recommended pandemic hygiene?** |  |  |  |
| All of the time | 925 (77.2) | 348 (77.7) | 568 (75.6) |
| Not all of the time | 269 (22.4) | 99 (22.1) | 180 (24.0) |
| Unknown | 5 (0.4) | 1 (0.2) | 3 (0.4) |
| **Social distancing causing stress in the past month** |  |  |  |
| A lot | 294 (24.5) | 94 (21.0) | 189 (25.2) |
| Somewhat | 357 (29.8) | 161 (35.9) | 219 (29.2) |
| A little | 304 (25.4) | 98 (21.9) | 191 (25.4) |
| Not at all | 238 (19.9) | 93 (20.8) | 147 (19.6) |
| Unknown | 6 (0.5) | 2 (0.5) | 5 (0.7) |

Supplemental Figure 1. Survey questions utilized in analysis.

**Q1. How much, if at all, has physically distancing yourself from others due to COVID-19 negatively affected your emotional or mental health?**

- A lot
- Some
- Just a little o Not at all
- Have not been physically distancing myself from others

**Q2. In the past month, have recommendations for social distancing caused stress for you?** o A lot o Somewhat

- A little
- Not at all

**Q3. In the past 7 days, how often have you....**

|  | Rarely or none of the time (less than 1 day) | Some or a little of the  time (1-2 days) | Occasionally or a moderate amount of  time (3-4 days) | Most or all of the time  (5-7 days) |
| --- | --- | --- | --- | --- |
| Felt nervous, anxious or on edge | o | o | o | o |
| Felt depressed | o | o | o | o |
| Felt lonely | o | o | o | o |
| Felt hopeful about the future | o | o | o | o |
| Had trouble sleeping | o | o | o | o |

**Q4. Please select one option for each of the following statements:**

|  | Strongly disagree | Disagree | Neutral | Agree | Strongly Agree |
| --- | --- | --- | --- | --- | --- |
| I am most afraid of Coronavirus | o | o | o | o | o |
| It makes me uncomfortable to think about Coronavirus | o | o | o | o | o |
| My hands become clammy when I think about  Coronavirus | o | o | o | o | o |
| I am afraid of losing my life because of Coronavirus | o | o | o | o | o |
| When I watch news and stories about Coronavirus  on social media, I become nervous or anxious | o | o | o | o | o |
| I cannot sleep because  I’m worrying about getting Coronavirus | o | o | o | o | o |
| My heart races or palpitates when I think about getting Coronavirus | o | o | o | o | o |

**Q5. How often in the past month are you doing the recommended pandemic hygiene, like washing hands frequently, avoiding touching your face, covering coughs, and avoiding frequently touched surfaces in public places?**

- All of the time o Most of the time o Sometimes
- Rarely

**Q6. What is your main source of employment right now?** o Employed full time by someone else (> 32 hours/week) o Employed part time by someone else (1-31 hours/week) o Self-employed, I own my own business o Not employed, but looking for work o Not employed

- Retired
- Disabled, not able to work
- Student
- Other

**Q7. Are you considered to be at higher risk for severe illness if you were to be diagnosed COVID-19?**

- Yes
- No
- I don’t know

**Q8. Are there any health conditions that you are currently or should be in ACTIVE treatment for?**

*Please select all that apply or select "no additional health conditions".*

| ▢ | Arthritis | |
| --- | --- | --- |
| ▢ | Asthma | |
| ▢ | Autoimmune disease | |
| ▢ | Cancer | |
| ▢ | Cardiovascular disease | |
| ▢ | Colitis | |
| ▢ | Crohn’s disease | |
| ▢ | Chronic Fatigue Syndrome | |
| ▢ | Chronic pain | |
| ▢ | Chronic obstructive pulmonary disease (COPD) | |
| ▢ | Cystic fibrosis | |
| ▢ | Diabetes | |
| ▢ | Epilepsy | |
| ▢ | Genetic disorder | |
| ▢ | Heart disease | |
| ▢ | Hepatitis | |
| ▢ | HIV/AIDS | |
| ▢ | Hypertension | |
| ▢ | Kidney disease | |
| ▢ | Lupus | |
| ▢ | Migraine headaches | |
| ▢ | Multiple sclerosis | |
| ▢ | Myelodysplastic syndrome | |
| ▢ | Nervous system condition | |
| ▢ | Neuromuscular disease | |
| ▢ | Osteoporosis | |
| ▢ | Pulmonary condition |  |
| ▢ | Sickle Cell Anemia |  |
| ▢ | Vascular disease |  |
| ▢ | Other chronic disease or illness |  |
| ▢ | No additional health conditions |  |

**Q9. Please select the type of cancer you were diagnosed with from the list below:**

- Bladder o Bone o Breast o Colon or Rectal o Endocrine o Gastrointestinal o Genitourinary o Gynecologic o Head and Neck o Hodgkin Lymphoma o Leukemia o Liver o Lung o Myeloma o Neurological o Non-Hodgkin Lymphoma
- Ocular o Prostate o Sarcoma o Skin o Thyroid
- Other ________________________________________________

**Q10. Have you experienced any disruption in your ability to access necessary health care for this condition since February because you had to cancel an appointment and/or could not get an appointment for any of the following?**

Please select all that apply.

| ▢ | Primary care physician |  |
| --- | --- | --- |
| ▢ | Specialty care doctors (oncologist, infectious disease specialist etc.) |  |
| ▢ | Laboratory or diagnostic testing |  |
| ▢ | Surgery or surgical procedures |  |
| ▢ | Physical therapy |  |
| ▢ | Mental health |  |
| ▢ | Occupational therapy | |
| ▢ | Dental services | |
| ▢ | Ongoing medical services like chemotherapy or dialysis administered at a clinic | |
| ▢ | Other healthcare services | |
| ▢ | I have not had trouble | |

**Q11. Thinking only about the health condition(s) you just mentioned, have you delayed care or had your treatment interrupted due to the COVID-19 pandemic?**

- I chose to delay or postpone care/treatment due to COVID-19 o Care/treatment was delayed because my hospital/provider did not allow it (or could not treat you) due to COVID-19 restrictions
- Care/treatment was interrupted because of loss of income related to COVID-

19 o Care/treatment was delayed due to difficulty accessing medications or other medical care due to shelter in place or stay home orders

- Care/treatment was interrupted because of loss of health insurance related to

COVID-19 o I did not experience any delay in treatment or interruption in care

**Q12. To what extent are you concerned about long term health issues related your delay in care for your other health condition(s)?**

- Extremely concerned o Very concerned o Moderately concerned o Slightly concerned
- Not concerned at all

**Q13. Have you changed the way you access care? Or changed where/how your receive it?**

**Please select all that apply.**

▢ Needed to use alternate or out of network providers

▢ Utilized telehealth instead of in-office

▢ Distance traveled to care has increased

▢ Use an online pharmacy/changed how medications are accessed (i.e. 3 month vs 1 month supply)

▢ Cancelled routine care for other chronic diseases

▢ Utilized in home health care instead of in office

▢ Delayed care or medical procedures

▢ No changes to care

Supplemental Figure 2. Comparison of disruption in ability to access necessary health care for comorbidities due to cancelled appointment or inability to get an appointment for all respondents who completed the first survey with more fear of COVID-19 (n=464) and less fear of COVID-19 (n=735).

0

%

%

5

%

10

15

%

20

%

25

%

30

%

35

%

40

%

Unknown

Occupational therapy

Ongoing medical services from a clinic

Physical therapy

Other healthcare services

Mental health

Surgery or other surgical procedures

Laboratory or diagnostic testing

Dental services

Primary care physician

Specialty care doctors

All first survey, more fear of COVID-19 (n=464)

All first survey, less fear of COVID-19 (n=735)

**Respondents were able to report multiple options.*

Supplemental Figure 3. Comparison of change in the way care is accessed for all respondents who completed the first survey with more fear of COVID-19 (n=464) and less fear of COVID-19 (n=735).

%

0

%

5

%

10

%

15

%

20

25

%

30

%

35

%

40

%

45

%

%

50

Unknown

Needed to use alternate or out of network providers

Utilized in home health care

Increased distance to care

Changed how accessed medications

Cancelled routine care

Delayed care or procedures

Utilized telehealth

All first survey, more fear of COVID-19 (n=464)

All first survey, less fear of COVID-19 (n=735)

**Respondents were able to report multiple options.*
